# CcpA Regulates Arginine Biosynthesis in *Staphylococcus aureus* through Repression of Proline Catabolism

**DOI:** 10.1371/journal.ppat.1003033

**Published:** 2012-11-29

**Authors:** Austin S. Nuxoll, Steven M. Halouska, Marat R. Sadykov, Mark L. Hanke, Kenneth W. Bayles, Tammy Kielian, Robert Powers, Paul D. Fey

**Affiliations:** 1 Department of Pathology and Microbiology, University of Nebraska Medical Center, Omaha, Nebraska, United States of America; 2 Department of Chemistry, University of Nebraska-Lincoln, Lincoln, Nebraska, United States of America; Harvard Medical School, United States of America

## Abstract

*Staphylococcus aureus* is a leading cause of community-associated and nosocomial infections. Imperative to the success of *S. aureus* is the ability to adapt and utilize nutrients that are readily available. Genomic sequencing suggests that *S. aureus* has the genes required for synthesis of all twenty amino acids. However, *in vitro* experimentation demonstrates that staphylococci have multiple amino acid auxotrophies, including arginine. Although *S. aureus* possesses the highly conserved anabolic pathway that synthesizes arginine via glutamate, we demonstrate here that inactivation of *ccpA* facilitates the synthesis of arginine via the urea cycle utilizing proline as a substrate. Mutations within *putA*, *rocD*, *arcB1*, *argG* and *argH* abolished the ability of *S. aureus* JE2 *ccpA::tetL* to grow in the absence of arginine, whereas an interruption in *argJBCF*, *arcB2*, or *proC* had no effect. Furthermore, nuclear magnetic resonance demonstrated that JE2 *ccpA::ermB* produced ^13^C_5_ labeled arginine when grown with ^13^C_5_ proline. Taken together, these data support the conclusion that *S. aureus* synthesizes arginine from proline during growth on secondary carbon sources. Furthermore, although highly conserved in all sequenced *S. aureus* genomes, the arginine anabolic pathway (ArgJBCDFGH) is not functional under *in vitro* growth conditions. Finally, a mutation in *argH* attenuated virulence in a mouse kidney abscess model in comparison to wild type JE2 demonstrating the importance of arginine biosynthesis *in vivo* via the urea cycle. However, mutations in *argB*, *argF*, and *putA* did not attenuate virulence suggesting both the glutamate and proline pathways are active and they, or their pathway intermediates, can complement each other *in vivo*.

## Author Summary

Although *Staphylococcus aureus* encodes the highly conserved arginine biosynthesis pathway via glutamate, arginine is an essential amino acid. We found that a mutation in *ccpA*, a gene encoding a protein facilitating carbon catabolite repression, mediates arginine biosynthesis under *in vitro* growth conditions. However, both genetic and biochemical evidence suggested that a *S. aureus ccpA* mutant synthesizes arginine via proline and the urea cycle, a pathway not demonstrated in bacteria before. Furthermore, an animal model of *S. aureus* infection demonstrated the importance of arginine biosynthesis *in vivo*. This new pathway sheds light on important host-pathogen interactions and suggests *S. aureus* has evolved to address arginine depletion in the host by synthesizing arginine from a readily available substrate such as proline.

## Introduction

*Staphylococcus aureus* is a common cause of skin and soft tissue infections; however more serious complications such as bacteremia, osteomyelitis, endocarditis, and necrotizing pneumonia can occur [1]. During infection, *S. aureus* must catabolize diverse carbon sources including carbohydrates, proteins and lipids; therefore, multiple global regulators, including CcpA and CodY, subsequently regulate carbon flow [2], [3], [4]. Thus, regulation of carbon flow through central metabolism and other metabolic pathways has a direct link to expression and synthesis of virulence factors [5], [6], [7].

It has been known for over 70 years that *S. aureus* exhibits multiple amino acid auxotrophies, including arginine, valine, proline, cysteine, and leucine [8], [9]. Complicating the picture, in 1937, Gladstone demonstrated that multiple strains of *S. aureus* could be trained to grow in a chemically-defined broth lacking all twenty amino acids through extended incubation [9]. These data suggested that *S. aureus* was indeed a prototroph but repressed biosynthesis of certain amino acids. In support of this, bioinformatic analyses of the *S. aureus* genome revealed an apparently complete repertoire of biosynthetic operons needed to synthesize all 20 amino acids [10]. Included in these are the genes encoding the arginine biosynthetic pathway *argJBCDFGH* where arginine is synthesized from glutamate [11]. This pathway is highly conserved among a wide array of bacteria, including *Escherichia coli*, *Salmonella enterica* serotype Typhimurium, *Proteus mirabilis*, *Bacillus subtilis*, and *Streptomyces clavuligerus* among others [12], [13], [14].

Although *B. subtilis* synthesizes proline from glutamate [11], [15], [16], *S. aureus* preferentially utilizes arginine rather than glutamate as a precursor for proline biosynthesis via arginase (RocF), ornithine aminotransferase (RocD), and P5C reductase (ProC) [17]. Furthermore, Li and colleagues recently reported that proline biosynthesis is regulated through CcpA-mediated carbon catabolite repression at both *rocF* and *rocD* [18]. Carbon catabolite repression allows bacteria to preferentially utilize preferred carbon sources and therefore increase the organism's fitness [19]. The *trans*-acting carbon catabolite protein CcpA in a complex with Hpr binds to *cis*-acting DNA sequences known as catabolite responsive elements (CRE) [20], [21], [22], [23]. In the presence of a preferred carbon source, HprK phosphorylates the Ser-46 position of Hpr and once phosphorylated, Hpr binds to CcpA [23], [24], [25].

In this study, we utilized genetic and biochemical approaches to examine arginine auxotrophy in *S. aureus*. *bursa aurealis* transposon mutagenesis identified CcpA as a regulator of arginine biosynthesis. However, instead of de-repressing the conserved arginine biosynthesis pathway (ArgJBCDFGH) via glutamate, *S. aureus* JE2 *ccpA* synthesized arginine from proline via the urea cycle. To the best of our knowledge, this is the first report of bacteria utilizing proline for arginine biosynthesis, which may indicate a predilection to degrade and utilize proteins rich in proline (i.e. collagen) during an *S. aureus* infection for use in arginine biosynthesis. Utilization of proline to synthesize arginine demonstrates the resourcefulness of *S. aureus* and its ability to rapidly evolve to utilize nutrients that are readily available in the environment.

## Results

### Arginine Auxotrophy in *Staphylococcus aureus*

To examine arginine auxotrophy in *S. aureus*, *e*ighty-two clinical *S. aureus* isolates collected from positive blood cultures at the University of Nebraska Medical Center were grown on Complete Defined Medium (CDM) with and without arginine. Similar to observations by Emmett and Kloos, only one *S. aureus* isolate (SA2126) had the ability to grow on CDM lacking arginine (CDM-R) following 48 h incubation, whereas all isolates grew on CDM containing arginine further confirming the arginine auxotrophic nature of *S. aureus* [8]. Furthermore, a community-associated *S. aureus* USA300 strain JE2 (Table S1) was unable to grow on CDM-R following 48 h incubation at 37°C. To extend these observations, JE2 was grown to stationary phase in CDM broth (5×10^9^ CFU) and plated on CDM-R and CDM lacking proline (CDM-P). Similar to the observations of Li and colleagues [18], *S. aureus* JE2 reverted to proline prototrophy at a rate of 1×10^−6^; however, no colonies were isolated on CDM-R following five experimental attempts. Nevertheless, similar to observations by Gladstone, slight growth of JE2 was observed following five days of incubation in CDM-R broth [9]. These observations suggest that *S. aureus* has the inherent ability to synthesize arginine upon extended selection; however, the phenotype is not easily selected during growth in medium replete with amino acids.

### Regulation of Arginine Biosynthesis by Carbon Catabolite Repression

**Figure 1:**
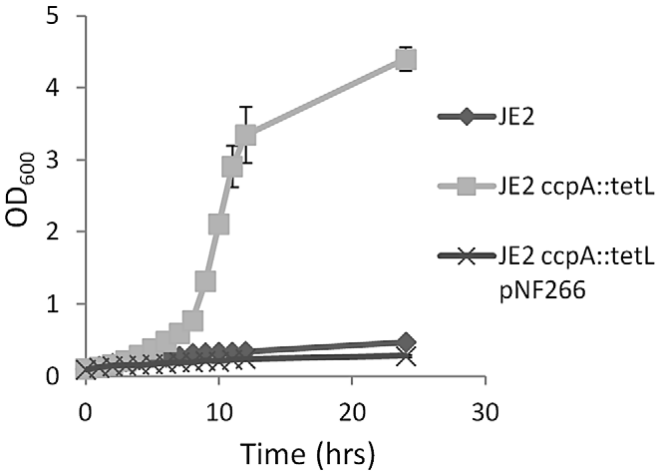
Interruption of *ccpA* facilitates growth in CDM-R. Growth analysis of JE2, JE2 *ccpA::tetL*, and JE2 *ccpA::tetL/*pNF266 (*ccpA* complement) in complete defined medium lacking arginine (CDM-R). Isolates were grown aerobically using a 10∶1 flask to volume ratio. *S. aureus* strains containing a functional *ccpA* are unable to grow in CDM-R. Data represent means ± SEM of three independent experiments.

Based on our observations that growth in CDM-R could be selected through extended incubation, we hypothesized that arginine biosynthesis was under transcriptional repression. Therefore, we screened a random *bursa aurealis* transposon library to isolate JE2 mutants able to grow on CDM-R. Two mutants were isolated that had the ability to grow on CDM-R; subsequent sequencing of the *bursa aurealis* insertions found they had inserted in *hprK* and *ccpA*. Both HprK and CcpA function to control carbon catabolite repression (CCR) in gram-positive bacteria [26]. Therefore, to completely eliminate CCR, a *ccpA* allelic replacement mutant was generated in JE2 through 80α transduction of the *ccpA::tetL* allele from MST14 (kind gift of M. Bischoff). As predicted, growth analysis in CDM-R demonstrated that JE2 *ccpA::tetL* enters exponential phase between 7–12 h and reaches a maximum OD_600_ of 4.5 after 24 h, whereas no growth was observed with wild type JE2 in CDM-R (Figure 1). Importantly, introduction of the *ccpA* complementation plasmid pNF266 abrogated growth of JE2 *ccpA::tetL*.

**Figure 2:**
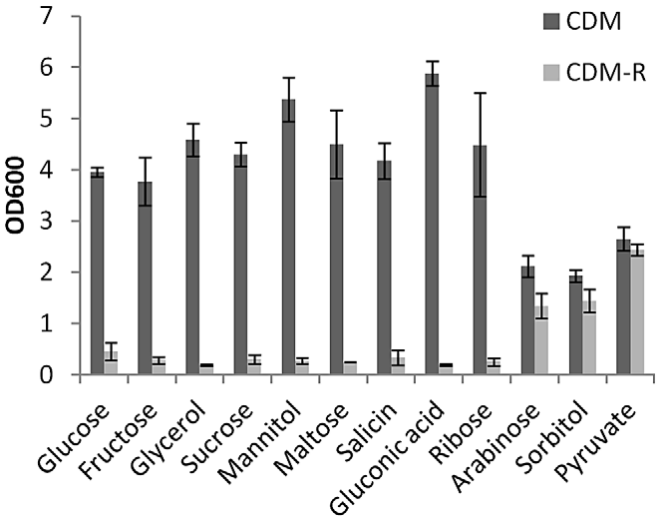
Growth of JE2 in CDM containing non-preferred carbon sources. JE2 was grown in CDM or CDM-R with the indicated carbon source at 37°C for 18 hours. Significant growth in CDM-R was seen only when arabinose and sorbitol were used as carbon sources. Data represent means ± SEM of five independent experiments.

To further support the hypothesis that CCR functions to repress arginine biosynthesis, JE2 was grown in CDM-R lacking glucose but containing other, non-preferred carbon sources (Figure 2). Since CCR is alleviated when *S. aureus* is grown in media containing a non-preferred carbon source, it was hypothesized that JE2 would grow in CDM-R when glucose was replaced with a secondary carbon source. These experiments demonstrated that arabinose, sorbitol and pyruvate were able to support growth of JE2 when added to CDM-R (Figure 2). In contrast, glucose, fructose, glycerol, sucrose, mannitol, maltose, salicin, gluconic acid, and ribose were unable to support growth in CDM-R suggesting these carbohydrates do not derepress CcpA in JE2. In agreement with our results, Li and colleagues also determined that replacement of glucose with arabinose or sorbitol abrogated CcpA-mediated repression in *S. aureus* Newman and functioned to activate proline biosynthesis [18]. Overall, these data demonstrate that CCR functions to repress arginine biosynthesis, suggesting that arginine biosynthesis is linked to growth in niches where preferred carbon sources are limited.

### Northern Analysis of *argJBCDFGH* in JE2 *ccpA::ermB*

**Figure 3:**
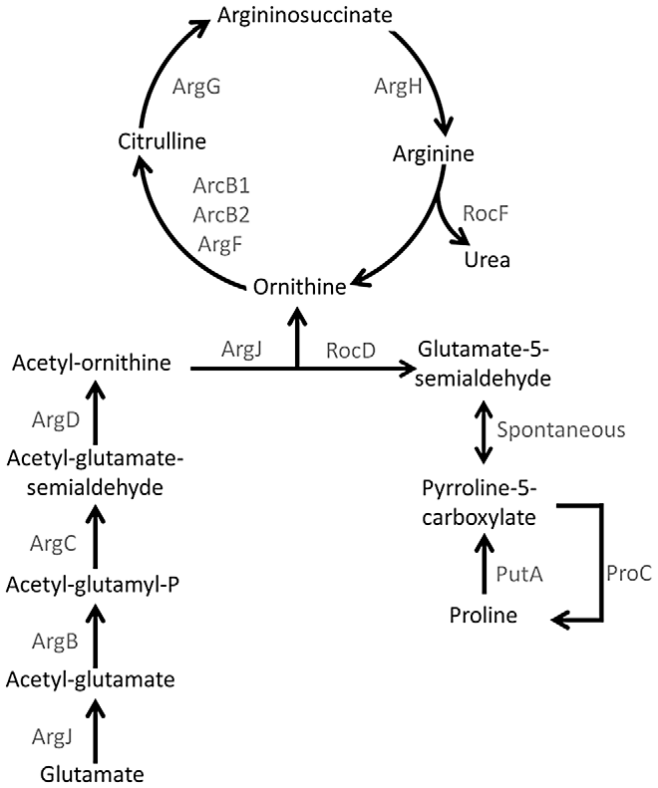
Arginine biosynthetic pathway via glutamate and proline. Figure depicts highly conserved arginine biosynthetic pathway via glutamate and the proposed pathway from proline via PutA, RocD, ArcB1, ArgG and ArgH. Note the previously established reverse pathway from arginine to proline via RocF, RocD and ProC.

**Figure 4:**
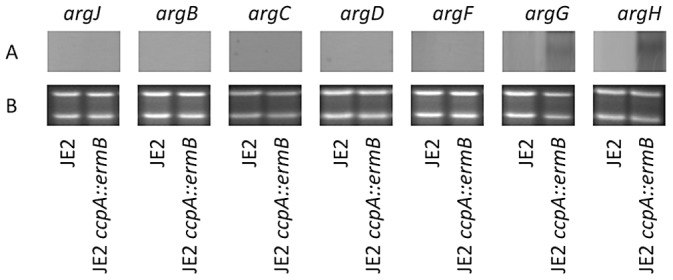
Northern analysis of arginine biosynthetic pathway in *S. aureus* JE2 and JE2 *ccpA::ermB*. JE2 and JE2 *ccpA::ermB* total RNA was isolated in mid-exponential phase of growth in CDM and CDM-R, respectively. DNA probes specific for *argJ*, *argB*, *argC*, *argD*, *argF*, *argG*, and *argH* were labeled with digoxygenin and detected using anti-digoxigenin-AP Fab fragments (Panel A). Panel B shows 16 s and 23 s rRNA depicting equal RNA loading.

Our preliminary data suggested that CCR functioned to repress an enzymatic step in the conserved arginine biosynthetic pathway via glutamate [27] (Figure 3). To further address this possibility, northern blot analysis was performed to address transcriptional expression of *argJBCDFGH* in JE2 *ccpA::ermB* in comparison to wildtype JE2. In *S. aureus*, *argDCJB* is arranged in an operon structure, whereas *argF* is transcribed as a monocistronic unit and *argGH* are co-transcribed. JE2 and JE2 *ccpA::ermB* were grown in CDM and CDM-R, respectively, to mid-exponential phase and mRNA was isolated. Utilizing DNA probes specific for each gene within the conserved pathway, *argDCJB* and *argF* expression was not detected in either JE2 or JE2 *ccpA::ermB* (Figure 4). However, although *argG* and *argH* transcripts were not detected in JE2, both transcripts were detected in JE2 *ccpA::ermB* (Figure 4). Therefore, although JE2 *ccpA::ermB* has the ability to grow on media lacking arginine, this strain does not appear to utilize the conserved arginine biosynthetic operon to synthesize arginine in CDM-R. These results suggested the existence of a novel arginine biosynthetic pathway in *S. aureus*.

### *S. aureus* Utilizes a Novel Proline Catabolic Pathway to Synthesize Arginine

Since our data indicated that glutamate was not the precursor for arginine synthesis in JE2 *ccpA::ermB*, other potential pathways were examined *in silico*. Based on the northern blot data demonstrating the expression of *argGH* in JE2 *ccpA::ermB*, we hypothesized that arginine may be synthesized via the urea cycle (Figure 3). *In silico* analysis predicted that either glutamate or proline have the potential to feed into the urea cycle to serve as substrates for arginine biosynthesis. To address this hypothesis, we examined amino acid consumption by JE2 and JE2 *ccpA::ermB* grown in CDM and CDM-R, respectively (Figure S1). These results demonstrated that both JE2 and JE2 *ccpA::ermB* consumed similar amounts of glutamate from the culture media following 24 h of growth. In contrast, JE2 *ccpA::ermB* consumed all available free proline from the culture medium, whereas only approximately 50% of the available free proline was consumed by JE2. Taken together, these observations allowed us to speculate that JE2 *ccpA::ermB* utilized proline via the urea cycle for arginine synthesis.

**Figure 5:**
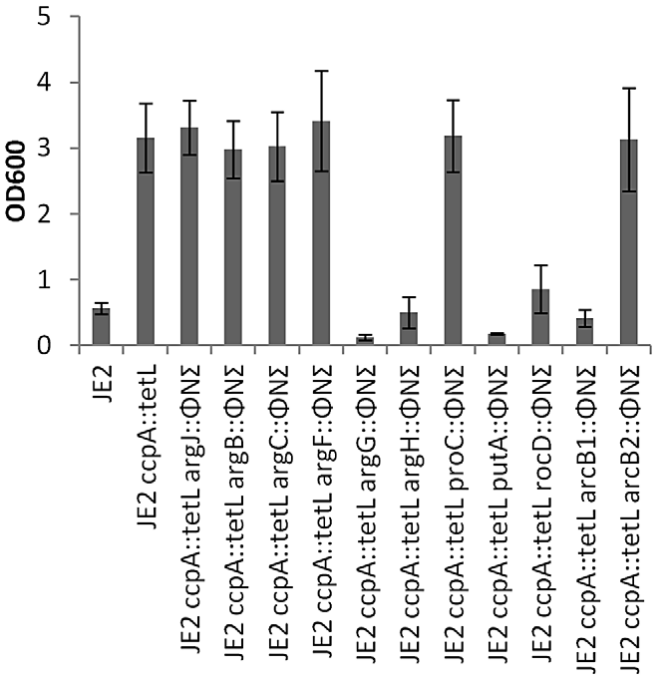
Determination of arginine biosynthesis-dependent genes in *S. aureus* JE2 *ccpA::tetL*. Defined *bursa aurealis* transposon mutants in *argJ*, *argB*, *argC*, *argF*, *argG*, *argH*, *proC*, *putA*, *rocD*, *arcB1*, and *arcB2* were transduced into JE2 *ccpA::tetL* and assessed for growth in CDM-R for 18 hours. Data represent means ± SEM of three independent experiments.

To further investigate this hypothesis, φ11 transducing lysates were prepared from defined JE2 *bursa aurealis* mutants with insertions in the following genes: *putA*, *proC*, *rocD*, *arcB1*, *arcB2*, *argF*, *argG*, *argH*, *argC*, *argB* and *argJ*. These *bursa aurealis* mutations (conferring erythromycin resistance) were transduced into JE2 *ccpA::tetL* and subsequently grown in CDM-R (Figure 5). Mutations in *argG*, *argH*, *putA*, *rocD*, and *arcB1* abrogated growth of JE2 *ccpA::tetL* in CDM-R. However, mutations in *argJ*, *argB*, *argC*, *argF*, *arcB2*, or *proC* had no effect on growth consistent with the prediction that arginine is synthesized from proline and not glutamate (Figure 5).

**Figure 6:**
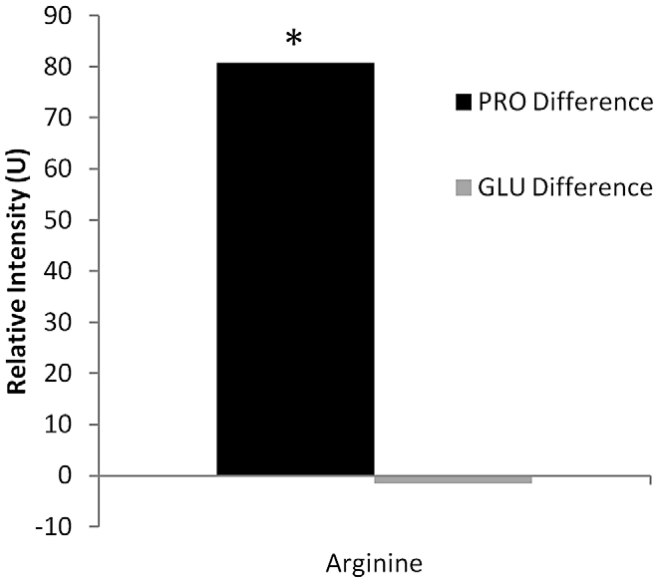
Two-dimensional (2D) ^1^H-^13^C heteronuclear single quantum coherence (HSQC) nuclear magnetic resonance (NMR) analysis of JE2 and JE2 *ccpA::ermB*. JE2 and JE2 *ccpA::ermB* were grown in the presence of ^13^C_5_-glutamate or ^13^C_5_-proline in CDM and CDM-R, respectively, and assayed using 2D ^13^C HSQC NMR. The differences in ^13^C-arginine relative intensity were determined by subtracting the average intensities between JE2 and JE2 *ccpA::ermB*, and a student's t-test was utilized to determine significance. A positive relative intensity value is indicative of a greater intensity of ^13^C-arginine in JE2 *ccpA::ermB* in comparison to JE2. JE2 *ccpA::ermB* accumulated significantly greater amounts of ^13^C-arginine when grown in CDM containing ^13^C_5_-proline in comparison to JE2. Note that there was no significant difference in ^13^C-arginine accumulation between JE2 and JE2 *ccpA::ermB* when grown in CDM and CDM-R, respectively, containing ^13^C_5_-glutamate.

Two-dimensional (2D) ^1^H-^13^C heteronuclear single quantum coherence (HSQC) nuclear magnetic resonance (NMR) experiments were performed to confirm these data. JE2 and JE2 *ccpA::ermB* were grown in the presence of ^13^C_5_-glutamate or ^13^C_5_-proline in CDM and CDM-R, respectively. Based on our genetic studies, it was predicted that ^13^C-labeled arginine would only be detected when JE2 *ccpA::ermB* was grown in CDM-R containing ^13^C_5_-proline. As expected, ^13^C-labeled arginine was detected when JE2 *ccpA::ermB* was propagated in the presence of ^13^C_5_-proline but not with ^13^C_5_-glutamate (Figure 6). Collectively, these results provide strong evidence that proline is the substrate for arginine biosynthesis in a *ccpA* genetic background. Furthermore, it is demonstrated that the highly conserved arginine biosynthetic pathway via glutamate is inactive under the growth conditions utilized in the study.

### Arginine Auxotrophy in Other *Staphylococcus aureus* Strains

**Figure 7:**
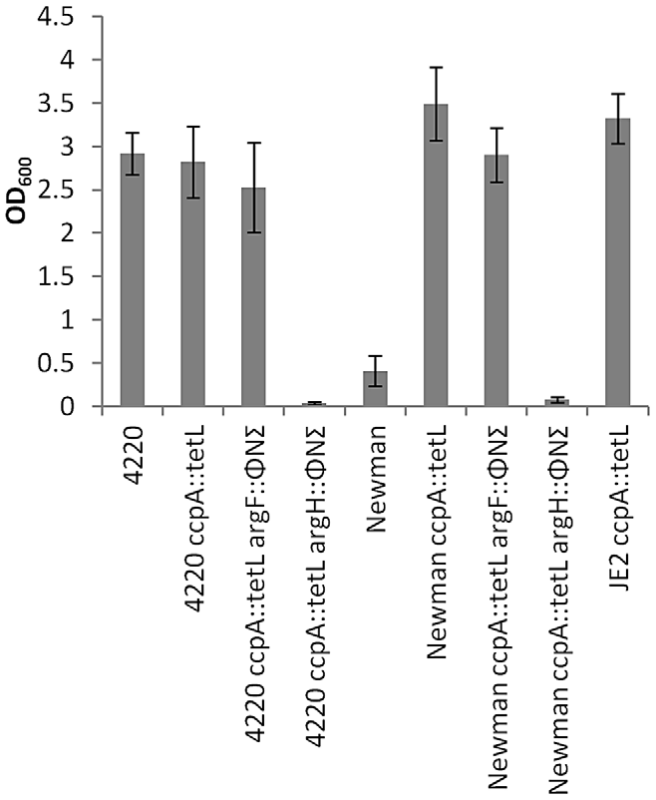
Determination of arginine biosynthesis-dependent genes in *S. aureus* Newman and RN4220. Defined *bursa aurealis* transposon mutants in *argF* and *argH* were transduced into Newman *ccpA::tetL* and RN4220 *ccpA::tetL* and assessed for growth in CDM-R for 18 hours. Data represent means ± SEM of three independent experiments.

To determine whether our data regarding arginine biosynthesis were specific to the JE2 background, φ11 transducing lysates were prepared from JE2 *bursa aurealis argF* and *argH* mutants and introduced into RN4220 and Newman *ccpA* backgrounds. As previously noted with JE2 *ccpA::tetL*, an *argH* mutation abolished the ability of both RN4220 *ccpA::tetL* and Newman *ccpA::tetL* to grow in CDM-R, whereas a mutation in *argF* had no effect (Figure 7). Interestingly, RN4220 has the ability to grow in CDM-R broth. Subsequent studies demonstrated that RN4220 reverted to arginine prototrophy at a frequency of 1.6×10^−5^; however, sequence analysis of these mutants indicated they did not have mutations in *ccpA*, *hprK* or *ptsH* suggesting weak carbon catabolite repression in the RN4220 strain background. In addition, RN4220 *argH::*φΝΣ was unable to grow in CDM-R broth whereas a *bursa aurealis* mutation in *argF* had no effect on growth suggesting RN4420 synthesizes arginine from proline but not from glutamate. Collectively, these data suggest that as a species, *S. aureus* has evolved to synthesize arginine via proline when growing in conditions lacking a preferred carbon source.

#### Virulence in a mouse kidney abscess model

**Figure 8:**
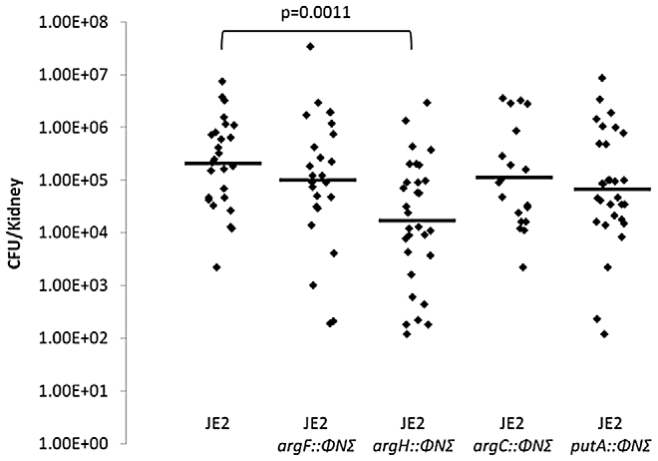
Mouse kidney abscess model. C57BL/6 mice were infected with 10^6^ CFU of JE2 (n = 16 mice), JE2 *argF::*φΝΣ (n = 14 mice), JE2 *argH::*φΝΣ (n = 15 mice), JE2 *argC::*φΝΣ (n = 13 mice), or JE2 *putA::*φΝΣ (n = 18 mice). Kidneys were homogenized after 20 days and bacterial burden determined through viable count (CFU/gram tissue). Horizontal line represents median log_10_ CFU/gram; significant differences in bacterial burden were noted between JE2 and JE2 *argH::*φΝΣ (**p<.01). Data were analyzed using two-way ANOVA.

C57BL/6 mice were inoculated retro-orbitally with 10^6^ CFU of JE2, JE2 *argH::*φΝΣ, JE2 *argC::*φΝΣ, JE2 *putA::*φΝΣ, or JE2 *argF::*φΝΣ. The mice were harvested at 20 days and the kidneys were homogenized and CFU/gram of tissue determined (Figure 8). No statistical difference was determined between JE2 and JE2 *argF::*φΝΣ, JE2 *argC::*φΝΣ, or JE2 *putA::*φΝΣ. However, a significant difference was noted between JE2 (mean log_10_ CFU of 5.31) and JE2 *argH::*φΝΣ (mean log_10_ CFU of 4.21) indicating a potential function of *argH* and arginine biosynthesis in abscess development and persistence.

## Discussion

The study of arginine biosynthesis has served as a paradigm for the regulon concept, originally coined by Maas and Clark, where the same transcriptional repressor regulates unlinked loci, ArgR [28]. Based on these studies and over 60 years of research, there are three established biochemical pathways, all utilizing glutamate as a substrate, that synthesize arginine in bacteria [27]. These three pathways primarily differ in the enzymes used to generate L-citrulline from N-acetyl-L-ornithine [27], [29]. All sequenced staphylococcal species analyzed to date encode the highly conserved ArgJBCDFGH on three separate unlinked transcriptional units; two operons (*argDCJB* and *argGH*) and one monocistronic gene (*argF*). Within the *S. aureus* USA300 FPR_3757 genome (NC_007793), the genes predicted to encode the arginine biosynthetic pathway are as follows: *argJ* (bifunctional ornithine acetyltransferase/glutamate N-acetyltransferase), SAUSA300_0185, EC 2.3.1.35/2.3.1.1; *argB* (acetylglutamate kinase), SAUSA300_0184, EC 2.7.2.8; *argC* (N-acetyl-gamma-glutamyl-phosphate-reductase), SAUSA300_0186, EC 1.2.1.38; *argD* (acetylornithine transaminase), SAUSA300_0187, EC 2.6.1.11; *argF* (ornithine carbamoyl transferase), SAUSA300_1062, EC 2.1.3.3; *argG* (argininosuccinate synthase), SAUSA300_0864, EC 6.3.4.5; and *argH* (argininosuccinate lyase), SAUSA300_0863, EC 4.3.2.1. However, as previously reported and confirmed in this study, *S. aureus* is a functional arginine auxotroph when grown on complex laboratory media [8], [9]. In addition, no nonsense mutations or insertions were detected within the *argJBCDFGH* genes of the USA300 FPR_3757 genome or any other sequenced staphylococcal genome, suggesting that arginine biosynthesis is not a decaying pathway in the staphylococci. As discussed by Somerville and Proctor, in some cases, amino acid auxotrophies in *S. aureus* may be linked to TCA cycle inactivity or feedback inhibition due to growth in amino acid and glucose replete media [30]. Our results are in agreement with this hypothesis where inactivation of *ccpA*, which represses the TCA cycle [31] and other genes that function to metabolize secondary carbon sources, was linked to arginine biosynthesis in *S. aureus*. In the presence of a preferred carbon source, the CcpA/Hpr complex represses a multitude of genes linked to central metabolism, amino acid metabolism and virulence [2], [32], [33], [34]. Therefore, based on previous studies, bioinformatic analyses of the *S. aureus* genome, and the work by Li and colleagues demonstrating that proline biosynthesis was linked to *ccpA* regulation, it was not unexpected to discover that arginine biosynthesis was linked to carbon catabolite repression [5], [18], [30], [35]. However, it was remarkable to discover that *S. aureus* does not use the conserved *argJBCDFGH* pathway to synthesize arginine via glutamate. Rather, we provide both genetic and biochemical evidence in support of a novel biosynthetic pathway, whereby *S. aureus* utilizes proline as a substrate via the urea cycle. First, mutations within *putA*, *rocD*, *arcB1*, *argG*, and *argH*, but not *argJ*, *argB*, *argC*, or *argF*, abolished growth of a *ccpA* mutant on CDM-R, providing genetic evidence that proline serves as a precursor for arginine synthesis (Figure 5). It is important to note that inactivation of *arcB1* abrogated growth of JE2 *ccpA::tetL* whereas a mutation in *arcB2* did not. *arcB1* (SAUSA300_2569) encodes the native ornithine carbamoyltransferase within the arginine deiminase operon whereas *arcB2* (SAUSA300_0062; ornithine carbamoyltransferase) is within the ACME pathogenicity island encoded arginine deiminase operon [36]. These data suggest that ArcB1 and ArcB2 are not functionally redundant or are not expressed under the same growth conditions. *arcB2* transcript is not detected using northern analysis (data not shown) under *in vitro* growth conditions used in this study (CDM or CDM-R broth), however, it is unknown whether it is induced under other *in vivo* or *in vitro* growth conditions. Second, 2D ^1^H-^13^C HSQC NMR experiments provided compelling evidence that arginine is synthesized via proline and the urea cycle in a *S. aureus ccpA* mutant. Although there have been two reports demonstrating that proline is synthesized from arginine in *S. aureus* [17], [18], we are unaware of any reports demonstrating that arginine can be synthesized from proline. Li and colleagues demonstrated that CcpA binds to a *cre* site just upstream of *rocD*. Using the *cre* site from *pckA* as a consensus sequence [18], we identified potential *cre* sites upstream of *putA*, *arcB1*, and *argGH* (Figure S2). However, the function of these *cre* sites in regards to CcpA regulation has yet to be defined.

**Figure 9:**
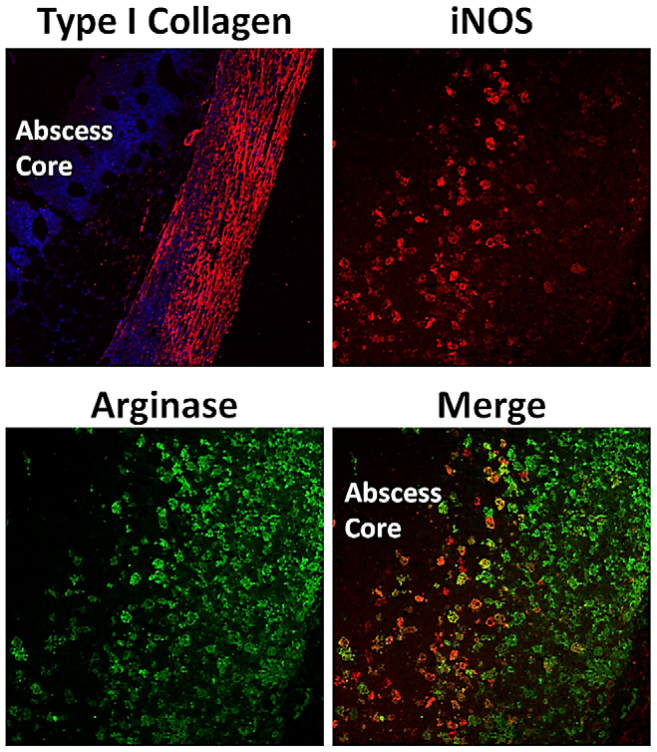
Mouse subcutaneous abscess model. C57BL/6 flank abscesses caused by subcutaneous injection of 5×10^5^ CFU of *S. aureus* JE2. Tissues were processed for immunofluorescence staining for either type I collagen (red), inducible nitric oxide synthase (iNOS, red), or arginase (green). In addition to type I collagen, tissues were processed with the DAPI nuclear stain (blue) to accentuate the abscess core. Representative confocal microscopy images are presented for type I collagen (10× magnification) and iNOS/arginase (20× magnification).

Previous studies have demonstrated that a *S. aureus ccpA* mutant also synthesizes proline from arginine via RocF (arginase), RocD (ornithine aminotransferase), and ProC (P5C reductase) [17], [18]. Collectively, these data and our observations suggest that under carbon-limiting conditions (*in vivo* environment), *S. aureus* can synthesize proline from arginine and arginine from proline depending on which amino acid is limited. Based on our findings and the existing literature, we propose a hypothetical model whereby free arginine is limited in the host during infection causing competition between the host and bacteria for arginine. In humans, L-arginine is a non-essential amino acid under homeostatic conditions. However, arginine becomes a “conditionally essential” [37] amino acid during sepsis or trauma due to its use as a substrate for inducible nitric oxide synthase [38] and function in cell-mediated immunity [39], protein synthesis [40] and wound healing [41], [42]. Indeed, recent studies have shown significant iNOS and arginase expression during *S. aureus* infection [43] (Figure 9), providing further support that a staphylococcal abscess may be an arginine-depleted environment based on the requirement of arginine for these host enzymes to function. In addition, low levels of L-arginine have been reported in plasma during sepsis [37], causing some investigators to suggest the use of L-arginine as a treatment modality [44]. Furthermore, arginine can serve as a substrate for arginine deiminase and subsequent direct ATP generation in the staphylococci [27].

Although little information is available regarding the concentration of free proline in a staphylococcal abscess, proline is the predominant amino acid found in collagen. Collagen is the most abundant protein in animals and type I collagen is a major constituent of the fibrotic wall surrounding staphylococcal abscesses [45] (Figure 9). Furthermore, *S. aureus* encodes two proteases, SspB and ScpA, which possess the ability to degrade collagen [46], [47], [48]. Therefore, our model predicts that *S. aureus* utilizes specific proteases to degrade collagen, resulting in the liberation of free proline or proline-containing peptides that are utilized to synthesize arginine via the urea cycle. Strengthening this argument, earlier work demonstrated that mutants lacking the high affinity proline permease PutP are less virulent in animal models of infection [49], [50]. This proposed framework was initially tested using a mouse kidney abscess model previously utilized by Cheng and colleagues [51]. In this model, staphylococcal abscesses within the kidney are contained within a pseudocapsule-like structure; we hypothesized an *argH* and *putA* mutant would be attenuated in abscess persistence in comparison to wild type JE2, JE2 *argC::*φΝΣ and JE2 *argF::*φΝΣ due to the inability to utilize proline from the pseudocapsule as a substrate for arginine synthesis. Supporting our model, in those kidneys containing staphylococcal abscesses, a significant 1 log_10_ difference was observed between JE2 and JE2 *argH::*φΝΣ demonstrating the importance of arginine biosynthesis via the urea cycle in an *in vivo* infection model. As predicted, based on our *in vitro* data, no significant difference was observed between JE2, JE2 *argC::*φΝΣ, and JE2 *argF::*φΝΣ in the mouse kidney abscess model. However, in contrast to our predicted results, no significant difference in bacterial persistence was detected between JE2 and JE2 *putA::* φΝΣ; PutA converts proline into pyrolline-5-carboxylate (Figure 3). It is known that the addition of either citrulline or ornithine to CDM-R can complement JE2 *ccpA::tetL putA::*φΝΣ allowing growth. Therefore, it is possible that generation of citrulline or ornithine by arginine deiminase and ornithine carbamoyltransferase [27], respectively, circumvents and complements the proline requirement and facilitates the synthesis of arginine. In addition, since *argGH* is common to both the glutamate and proline pathways leading to the synthesis of arginine, an alternative interpretation of the data is that both pathways are active *in vivo* and have the ability to complement each other.

Finally, we have demonstrated that other *S. aureus* strains synthesize arginine from proline when CcpA activity is abolished, suggesting conservation of this pathway within the species. However, based on the conserved sequence analysis of the ArgJBCDFGH pathway within sequenced *S. aureus* isolates, we predict that this arginine biosynthetic pathway is active under growth conditions or niches that remain to be identified. Further work is required to dissect the evolving dogma regarding arginine metabolism and the relationship between the host and *S. aureus* in the “war for arginine” during infection.

## Materials and Methods

### Ethics

The clinical *S. aureus* strains used in this study originated from the University of Nebraska Medical Center. The Institutional Review Board at the University of Nebraska Medical Center is charged with reviewing all research involving human subjects. The clinical *S. aureus* strains utilized in the study were de-identified and analyzed anonymously and were therefore exempt from human research committee approval.

Animal experimentation was performed under a University of Nebraska Medical Center approved Institutional Animal Care and Use Committee (IACUC) Protocol to TK. The University of Nebraska Medical Center is accredited by the Association for Assessment and Accreditation of Laboratory Animal Care International (AALAC). In addition, all animals at the University of Nebraska Medical Center are maintained in accordance with the Animal Welfare Act and the DHHS “Guide for the Care and Use of Laboratory Animals.”

### Bacterial Strains and Culture Conditions

For determination of arginine auxotrophy, eighty-two *S. aureus* isolates were obtained from a previous collection testing the prevalence of heterogeneous vancomycin intermediate susceptibility [52]. Other bacterial strains used in the study are shown in Table S1. Defined *bursa aurealis* transposon mutants were acquired from the Nebraska transposon mutant library via the Network on Antimicrobial Resistance in *Staphylococcus aureus* (NARSA; http://www.narsa.net). Bacterial strains were grown in either Tryptic Soy Broth (TSB; Becton Dickinson, Franklin Lakes, NJ) or Complete Defined Medium (CDM) as previously described except containing 0.25% glucose [53]. Cultures were grown aerobically (1∶10 volume to flask ratio) at 37°C, 250 rpm unless otherwise stated. To train JE2 to grow on media lacking arginine, cultures were grown in CDM-R broth for 6 days, at which point the bacteria were inoculated to CDM-R agar. To study the reversion frequency of JE2, Newman and RN4220, the bacteria were grown for 20 hours in 3 mL of CDM. Cells were pelleted, resuspended in 0.9% NaCl, and diluted onto CDM, CDM-R, or CDM-P. After 72 hours the colonies were counted and reversion frequency was determined by taking the number of prototrophic revertants divided by total number of colonies plated on CDM.

To determine the growth characteristics in CDM-R containing various alternative carbon sources, JE2 was grown in 3 mL of CDM overnight, pelleted and resuspended in 0.9% NaCl. 3 mL of CDM and CDM-R supplemented with either 0.25% of glucose, fructose, glycerol, sucrose, mannitol, maltose, salicin, gluconic acid, arabinose, sorbitol, or ribose (all purchased from Sigma-Aldrich, St. Louis, MO) were inoculated in a 14 mL polypropylene round-bottom tube (Becton Dickinson) to an OD_600_ of 0.05. Cultures were grown for 18 h at 37°C to stationary phase.

### Screening of Random *bursa aurealis* Transposon Mutant Library

Random *bursa aurealis* transposon mutants were generated using plasmids pBursa and pFA545 and identified using inverse PCR as previously described [54]. Mutants were grown and collected in a 96 well format and pelleted and resuspended in 50 uL of 0.9% NaCl. 2 uL were plated on CDM and CDM-R and incubated at 37°C for 72 hours. Approximately 2700 mutants were screened; colonies that grew on CDM-R plates were confirmed by growing in CDM-R broth.

### Transduction, *ccpA* Mutant Construction and Complementation

*bursa aurealis* transposon mutations were moved to other strain backgrounds through transduction using phage 80α or φ11 as previously described [55]. All primers (Table S2) used for construction and confirmation of the *ccpA* mutation were generated based on the sequence of *S. aureus* strain Mu50 (NC_002758.2). The *ccpA* mutant was constructed by replacing a 0.6 kb internal region of the *ccpA* gene with an erythromycin resistance cassette (*ermB*) using the gene splicing by overlap extension (gene SOEing) technique [56]. *ermB* was amplified from pEC4 [57] using primers SAV1736-ermB-f and SAV1736-ermB-r, which contain sequences homologous to the *ccpA* gene. Primers BamHI-SAV1737-f and ermB-SAV1736-r were used for amplification of a 1.3 kb region upstream of the *ccpA* gene. Primers ermB-SAV1736-f and SacI-acuC-f were used to amplify a 1.7 kb region downstream of the *ccpA* gene. The resulting 4.1-kb PCR product contained BamHI and SacI sites that were used for cloning into pTS1-d [58] to generate plasmid pMRS44. Plasmid pMRS44 was used to construct *S. aureus* SA564 *ccpA*::*ermB* using the temperature shift protocol as previously described [59]. Allelic replacement of the internal region of the *ccpA* gene by the *ermB* cassette was verified by PCR using primers ermB-f, ermB-r, SAV1737-f and acuC-f. The *ccpA::ermB* mutation was subsequently moved to JE2 through phage 80α transduction and confirmed using primers noted above. For the *ccpA* complementation plasmid pNF266, *ccpA* was amplified from JE2 using primers 2250 and 2251 (Table S2), digested with SphI and BamHI, and cloned into pCN51 [60]. Note that two *ccpA* mutants were constructed in this study, JE2 *ccpA::ermB* and JE2 *ccpA::tetL*. JE2 *ccpA::tetL* was generated by phage 80α transduction of the *ccpA::tetL* allele from MST14 [2] so double mutants could be constructed using *ermB* as the second selectable marker.

### NMR Data Collection

JE2 and JE2 *ccpA::ermB* were grown in 50 mL CDM to stationary phase. JE2 and JE2 *ccpA::ermB* were subsequently inoculated to an OD600 of 0.05 in CDM containing 100 µM of either ^13^C_5_-glutamate or ^13^C_5_-proline (Isotec) and grown to stationary phase. Cultures were normalized to an OD_600_ of 2.0 and pelleted by centrifugation (3000 rpm, 20 minutes, 4°C). Pellets were subsequently washed in 10 mL of cold sterile water and resuspended in 1 mL cold sterile water. The Pellet was lysed using a bead beater (MP Biomedicals) and centrifuged for 15 minutes at 13,000 rpm at 4°C. This lysis step was repeated two more times and the pellet frozen in an ethanol/dry ice bath. The samples were then lyophilized, suspended in 600 uL of 50 mM phosphate buffer (pH = 7.2, uncorrected) in 99.8% D_2_O (Isotec), and transferred to 5 mm NMR tubes for analysis. The NMR spectra were collected on a Bruker 500 MHz Avance spectrometer equipped with a triple-resonance, Z-axis gradient probe. A BACS-120 sample changer with Bruker Icon software was used to automate the NMR data collection. The 2D ^1^H-^13^C HSQC spectra were collected with a standard Bruker pulse sequence (HSQCETGP), solvent presaturation and a relaxation delay of 1.5s. Each 2D ^1^H-^13^C HSQC spectrum was collected with a spectrum width of 4734.85 Hz and 2048 data points in the direct (^1^H) dimension; and a spectrum width of 13834.26 Hz and 64 data points in the indirect (^13^C) dimension. A total of 16 dummy scans and 128 scans were used to obtain each 2D ^1^H-^13^C HSQC spectra.

The spectra were processed using the NMRPipe software package [61]. The spectra were Fourier transformed, manually phased, and baseline corrected. The processed 2D ^1^H-^13^C HSQC spectra were then analyzed using NMRView [62] to assign chemical shifts and intensities to each peak. The chemical shift list were assigned to specific metabolites using the Human Metabalome Database [63], Madison Metabolomics Database [64], and Platform for Riken Metabolomics [65] with a tolerance level of 0.05 ppm and 0.40 ppm in the ^1^H and ^13^C chemical shifts respectively. The presence of metabolites and metabolomics pathways was verified using the Kyoto Encyclopedia of Genes and Genomes (KEGG) [66] and MetaCyc [67] databases. The quantification of metabolomic peak intensities were performed in a similar manner as previously described [68]. The relative percent concentration difference was determined by subtracting averages from the two cultures. A student T-test was performed to verify the significance at a 95% confidence level, of the relative percent concentration differences.

### RNA Isolation and Northern Blot Analysis

Cultures of *S. aureus* JE2 and JE2 *ccpA::ermB* were grown overnight in CDM, diluted to an OD_600_ of 0.05 into fresh CDM or CDM-R (1∶10 volume to flask ratio, 250 rpm), and grown at 37°C to an OD_600_ of 1.5 (mid-exponential growth). Cells were pelleted at 3000×g for 20 minutes at 4°C and resuspended in RLT buffer with 1% β-mercaptoethanol. Next, they were transferred to lysing matrix B tubes (MP Biomedicals) and processed in a FP120 FastPrep cell disrupter (MP Biomedicals) for 24 seconds at a setting of 6.0. The cells were pelleted at 13000 rpm at 4°C for 15 minutes; top-phase was combined with 500 uL of ethanol. The samples were then processed using an RNeasy mini kit, according to manufactures instructions (Qiagen, Inc.). Primers listed in Table S2 were used to make DNA probes that were subsequently labeled with digoxigenin-labeled dUTP (Roche). 5 ug of RNA was used for northern analysis that was performed using DIG buffers and washes (Roche). Anti-Digoxigenin-AP Fab fragments (Roche) was used with ECF substrate (GE Healthcare) for detection. Blots were visualized using the Typhoon FLA 7000 imaging system (GE Healthcare).

### Amino Acid Analysis

JE2 and JE2 *ccpA::ermB* were grown overnight in 50 mL (500 mL flask) of CDM. Cultures were inoculated to a starting OD_600_ of 0.05 in CDM (100 mL in 1 L flask, 250 rpm, 37°C) and grown for 5 hours. 500 uL of media was collected and pelleted for 5 minutes at maximum speed. Supernatant was collected and filtered through 3,000 MWCO Amicon Ultra centrifugal filters (Millipore) according to manufactures instructions. Amino acid analysis was performed by the Protein Structure Core Facility, UNMC, using a Hitachi L-8800.

### Animal Models

#### Mouse subcutaneous abscess model

Subcutaneous abscesses were established in C57BL/6 mice following the injection of 5×10^5^ cfu of *S. aureus* JE2. Tissues were collected at day 7 post-infection and processed for immunofluorescence staining for either type I collagen (Millipore, Billerica, MA), inducible nitric oxide synthase (Abcam, Cambridge, MA), or arginase (Santa Cruz, San Diego, CA.) For type I collagen, tissues were incubated with the nuclear stain DAPI to accentuate the abscess core. *Mouse kidney abscess model*. C57BL/6 mice were anesthetized using ketamine and xylazine and 100 µl containing 10^7^ CFU of *S. aureus* JE2, JE2 *argH::*φΝΣ, or JE2 *argF::*φΝΣ were inoculated retro-orbitally. On day 20 following inoculation, the animals were sacrificed and the kidneys were excised, homogenized, and subsequently plated for bacteriological analysis (CFU/g of tissue) on Trypticase soy agar (TSA). Only those kidneys containing greater than 100 CFU/g of tissue were statistically analyzed. Pairwise comparisons were conducted and differences were adjusted for multiple comparisons using the Tukey-Kramer method to maintain an overall alpha = .05 across all comparisons.

## Supporting Information

Figure S1 **Amino acid analysis of JE2 and JE2** ***ccpA::ermB*** **following growth in CDM.** JE2 and JE2 *ccpA::ermB* were grown in CDM for 18 hours and supernatant was collected and analyzed for amino acid content. Percent of proline and arginine remaining is shown suggesting more efficient utilization of proline by JE2 *ccpA::ermB* in comparison to JE2.
(TIF)

Figure S2 **Putative** ***cre*** **sites in arginine biosynthesis-dependent genes.** Using the *cre* site from *pckA* as a consensus sequence, putative *cre* sites were identified in *rocD*, *arcB1*, *putA*, *and argGH. cre* site from *pckA* is the top sequence whereas the putative *cre* site from the identified gene is the bottom sequence.
(TIF)

Table S1 **Bacterial Strains and Plasmids used in study.**
(DOCX)

Table S2 **Oligonucleotides used in study.**
(DOCX)
