# Supplementary material for: CcpA Regulates Arginine Biosynthesis in Staphylococcus aureus through Repression of Proline Catabolism
Source: PLoS Pathog. 2012 Nov 29;8(11):e1003033. doi: 10.1371/journal.ppat.1003033 (PMC3510247; doi:10.1371/journal.ppat.1003033)
Supplement: Table S1 — Bacterial Strains and Plasmids used in study. (DOCX) [file ppat.1003033.s003.docx]

**Table S1. Bacterial Strains and Plasmids used in study**

**Bacterial Strain or Plasmid Relevant Phenotype Source**

pFA545 Encodes pBursa transposase; Tet^r^ [1]

pBursa Encodes *bursa aurealis* transposon; Erm^r^, [1]

Cam^r^

pNF266 pCN51 ([60](#_ENREF_60)) with *ccpA* cloned into the SphI This study

and BamHI site using primers 2250 and

2251 removing *cadA;* Amp^r^, Erm^r^

pMRS44 Derivative of pTS1-d ([58](#_ENREF_58)) with *ccpA::ermB* This study

Amp^r^, Cam^r^, Erm^r^

*S. aureus* RN4220 Restriction deficient NCTC8325-4 [2]

*S. aureus* Newman *S. aureus* ATCC 25904 [3]

*S. aureus* MST14 Newman containing an allelic replacement [4]

mutation (*tetL*) in *ccpA.* Tet^r^

*S. aureus* LAC USA300 PFGE type; ST8. Erm^R^ Cam^R^ [5]

Tet^R^

**Bacterial Strain or Plasmid Relevant Phenotype Source**

*S. aureus* JE2 LAC cured of all 3 native plasmids; Erm^s^, This study

Cam^s^, Tet^s^

*S. aureus* JE2 *ccpA::tetL* Allelic replacement mutation in *ccpA;* This study

transduced from MST14; Tet^r^

*S. aureus* SA564 *ccpA::ermB* Allelic replacement mutation in *ccpA* This study

Erm^R^

*S. aureus* JE2 *ccpA::ermB ccpA* mutant; transduced from This study

SA564 *ccpA::ermB;* Erm^R^

*S. aureus* RN4220 *ccpA::tetL* Allelic replacement mutation in *ccpA:* This study

transduced from MST14; Tet^r^

JE2 *ccpA::tetL/argJ::*φΝΣ *bursa aurealis argJ* mutation in *ccpA::tetL* This study

background.

JE2 *ccpA::tetL/argB::*φΝΣ  *bursa aurealis argB* mutation in *ccpA::tetL* This study

background.

JE2 *ccpA::tetL/argC::*φΝΣ *bursa aurealis argC* mutation in *ccpA::tetL* This study

background.

**Bacterial Strain or Plasmid Relevant Phenotype Source**

JE2 *ccpA::tetL/argF::*φΝΣ *bursa aurealis argF* mutation in *ccpA::tetL* This study

background.

JE2 *ccpA::tetL/argG::*φΝΣ *bursa aurealis argG* mutation in *ccpA::tetL* This study

background.

JE2 *ccpA::tetL/argH::*φΝΣ *bursa aurealis argH* mutation in *ccpA::tetL* This study

background.

JE2 *ccpA::tetL/proC::*φΝΣ *bursa aurealis proC* mutation in *ccpA::tetL* This study

background.

JE2 *ccpA::tetL/putA::*φΝΣ *bursa aurealis putA* mutation in *ccpA::tetL* This study

background.

JE2 *ccpA::tetL/rocD::*φΝΣ *bursa aurealis rocD* mutation in *ccpA::tetL* This study

background.

JE2 *ccpA::tetL/arcB1::*φΝΣ *bursa aurealis arcB1* mutation in *ccpA::tetL* This study

background.

JE2 *ccpA::tetL/arcB2::*φΝΣ *bursa aurealis arcB2* mutation in *ccpA::tetL* This study

background.

**Bacterial Strain or Plasmid Relevant Phenotype Source**

RN4220 *argH::*φΝΣ *bursa aurealis argH* mutation This study

RN4220 *argF::*φΝΣ *bursa aurealis argF* mutation This study

RN4220 *ccpA::tetL/argG::*φΝΣ *bursa aurealis argG* mutation in *ccpA::tetL* This study

background.

RN4220 *ccpA::tetL/argH::*φΝΣ *bursa aurealis argH* mutation in *ccpA::tetL* This study

background.

Newman *ccpA::tetL/argG::*φΝΣ *bursa aurealis argG* mutation in *ccpA::tetL* This study

background.

Newman *ccpA::tetL/argH::*φΝΣ *bursa aurealis argH* mutation in *ccpA::tetL* This study

background.

1. Bae T*,* Banger AK, Wallace A, Glass EM, Aslund F, et al. (2004) *Staphylococcus aureus* virulence genes identified by bursa aurealis mutagenesis and nematode killing. Proc Natl Acad Sci U S A 101:12312-12317.

2. Kreiswirth BN*,* Lofdahl S, Betley MJ, O'Reilly M, Schlievert PM, et al. (1983) The toxic shock syndrome exotoxin structural gene is not detectably transmitted by a prophage. Nature 305:709-712.

3. Duthie ES & Lorenz LL (1952) Staphylococcal coagulase; mode of action and antigenicity. J Gen Microbiol 6:95-107.

4. Seidl K*,* Stucki M, Ruegg M, Goerke C, Wolz C, et al. (2006) *Staphylococcus aureus* CcpA affects virulence determinant production and antibiotic resistance. Antimicrob Agents Chemother 50:1183-1194.

5. Kennedy AD, Otto M, Braughton KR, Whitney AR, Chen L, et al.*,* (2008) Epidemic community-associated methicillin-resistant *Staphylococcus aureus*: recent clonal expansion and diversification. Proc Natl Acad Sci U S A 105:1327-1332.
