# Supplementary material for: CcpA Regulates Arginine Biosynthesis in Staphylococcus aureus through Repression of Proline Catabolism
Source: PLoS Pathog. 2012 Nov 29;8(11):e1003033. doi: 10.1371/journal.ppat.1003033 (PMC3510247; doi:10.1371/journal.ppat.1003033)
Supplement: Table S2 — Oligonucleotides used in study. (DOCX) [file ppat.1003033.s004.docx]

**Table S2. Oligonucleotides used in study**

**Primer name Sequence Relevant characteristic**

1868 TCTACAAGAACGCGATGTGC Forward primer used to amplify *argF*

1869 TTCACCCAATGTAGCAACCA Reverse primer used to amplify *argF*

1872 CAAAATCTATGGGGCAGAGC Forward primer used to amplify *argG*

1873 CCATGCAACATCGCATTTAC Reverse primer used to amplify *argG*

1874 TGCAACTATGCTTGCGAATC Forward primer used to amplify *argH*

1875 TGCTAGTTCCGTTGCATTTG Reverse primer used to amplify *argH*

1876 ACCCCCTACTTCAAGGCACT Forward primer used to amplify *argD*

1877 CGTCTTGAAAAGCTGCAACA Reverse primer used to amplify *argD*

1878 TAGGTATCGTTGGCGGTAGC Forward primer used to amplify *argC*

1879 CTCGATTTCCGGTTTGTGTT Reverse primer used to amplify *argC*

1880 ACACAAACATGGGTTGCTCA Forward primer used to amplify *argJ*

1881 GTTTCCCATTGTGGATGGTC Reverse primer used to amplify *argJ*

1882 AACACACGCTCATTGCAGAC Forward primer used to amplify *argB*

1883 AGGACAGCCATTTTCAATCG Reverse primer used to amplify *argB*

1929 TTGCAGCGCATGATCAAGGT Forward qRT-PCR; *argF*

1930 TTCCACACTGGTACGCCTGAA Reverse qRT-PCR; *argF*

1933 TGGGCATGGAGTCGTGAAGA Forward qRT-PCR; *argG*

1934 CTCTGGTGGCGCAGCATAAG Reverse qRT-PCR: *argG*

1935 ATCGAAGGCAGCATTGCACA Forward qRT-PCR; *argH*

1936 CCACCAGCATCACCGATACG Reverse qRT-PCR; *argH*

1937 TGGGAGCAAGTCGTTCCAGA Forward qRT-PCR; *argD*

1938 CAAGCGCTGCCGTCGATATT Reverse qRT-PCR; *argD*

1939 TCGTTGGCGGTAGCGGTTAT Forward qRT-PCR; *argC*

**Primer name Sequence Relevant characteristic**

1940 GGTGCTGGTGTCGCAAAGA Reverse qRT-PCR; *argC*

1941 CATCGTCTTTGGCAAGTGCAG Forward qRT-PCR; *argJ*

1942 CAGCTGTGCATCCAAACCACA Reverse qRT-PCR; *argJ*

1943 TGGCGGTGGCCCATTTATTA Forward qRT-PCR; *argB*

1944 CCCCAGTTGAAGCAACAGCA Reverse qRT-PCR; *argB*

2250 CCCGGG*GCATGC*TGAATTCCA Forward *ccpA;* contains

AGCATTTTATGATGA SphI* restriction site

2251 CCCGGG*GGATCC*TGAATTTATT Reverse *ccpA;* contains

TTGTAGTTCCTCGGTA BamH1* restriction site

2186 TCGCTAAATTTTTCCAAACAAAA Forward *ccpA*

2187 AAAGGCATTCTTCCAACACC Reverse *ccpA*

1888 TTTGGGCATATGTTAACGACAG Forward *hprK*

AAAAACTAGTT

1889 GGGGGATCCCTACTCCTCACTC Reverse *hprK*

TTATGACTG

1886 AATGTACATATGGAACAAAATTC Forward *ptsH*

ATATGTAATC

1887 ACAGGATCCTTTAGTCAATCCTT Reverse *ptsH*

CTTTTGATAAGAC

BamHI-SAV1737F CCaGGATCCACGTATCACGTA *ccpA* SOEing

AGTTGAAACCTGAAG

**Primer name Sequence Relevant characteristic**

ermB-SAV1736R CAACATGACGAATCCCTCCTTC *ccpA* SOEing

GCGCTTCTCTTGCTACATCATAT

ATAGTAAC

SAV1736-ermBF CTATATATGATGTAGCAAGAG *ccpA* SOEing

AAGCGCGAAGGAGGGATTCG

TCATGTTGG

SAV1736-ermBR CAATTCAATGTATCACCTAAT *ccpA* SOEing

TGAAGGCCGCGACTCATAGA

ATTATTTCCTCCCG

ermB-SAV1736F CGGGAGGAAATAATTCTATGA *ccpA* SOEing

GTCGCGGCCTTCAATTAGGTG

ATACATTGAATTG

SacI-AcuC-F GCAGAGCTCAGCAACAAGCGT *ccpA* SOEing

TTGATGATATTCG

SAV1737F GCAACAAAGGACCATTTAACGA *ccpA* mutant confirmation

TAATAC

**Primer name Sequence Relevant characteristic**

AcuC F GGTGGACTTGAAATATTCGCTA *ccpA* mutant confirmation

CAG

ermB F GGGTCAATCGAGAATATCGTCA *ccpA* mutant confirmation

ACTG

ermB R GCCCTTTACCTGTTCCAATTTCG *ccpA* mutant confirmation

____________________________________________________________________________

*Restriction site denoted in italics
